# Effect of the African Traditional Medicine, *Sutherlandia frutescens*, on the Bioavailability of the Antiretroviral Protease Inhibitor, Atazanavir

**DOI:** 10.1155/2013/324618

**Published:** 2013-12-12

**Authors:** Adrienne C. Müller, Michael F. Skinner, Isadore Kanfer

**Affiliations:** ^1^Division of Pharmaceutics, Faculty of Pharmacy, Rhodes University, P.O. Box 94, Grahamstown 6140, South Africa; ^2^Biopharmaceutics Research Institute, Rhodes University, P.O. Box 94, Grahamstown 6140, South Africa

## Abstract

The objective of this study was to investigate the effect of *Sutherlandia frutescens* (SF) on the bioavailability of atazanavir (ATV) in twelve healthy male subjects. During Phase I (Day 1), subjects ingested a single dose of ATV and blood samples were drawn before dose and at 0.5, 1.0, 1.5, 2.0, 2.5, 3.0, 3.5, 4.0, 5.0, 6.0, 9.0, 12, 18, and 24 hours after dose. From Day 3 to Day 14, a single dose of milled SF was administered twice daily to each subject. During Phase II, Day 15, subjects ingested single doses of ATV and SF. Blood samples were drawn as previously described. Plasma was harvested from blood samples and the concentration of ATV therein was determined. For each phase, the mean ATV plasma concentration-time profile was plotted and the means of AUC_0–24_ and *C*_max_ for ATV were computed. The geometric mean ratios and confidence intervals (CIs) for *C*_max_ and AUC_0–24 hr_ were 0.783 (0.609–1.00) and 0.801 (0.634–1.01), respectively. The CIs for both PK parameters fell below the limits of the “no-effect” boundary, set at 0.8–1.25, indicating that SF significantly reduced the bioavailability of ATV. This may potentially result in subtherapeutic plasma concentrations and thus reduced anti-HIV efficacy of ATV.

## 1. Introduction

The use of African traditional medicines (ATMs) by HIV/AIDS patients in South Africa is a common phenomenon [1–4]. Anecdotal reports detail the use of the indigenous South African plants, *Hypoxis hemerocallidea* (African potato) and *Sutherlandia frutescens* (SF) for the treatment of HIV/AIDS [5]. Despite the widespread use of ATMs, the impact of these medicines on the safety and efficacy of antiretrovirals (ARVs) when used concurrently has not yet been fully determined.

The protease inhibitor (PI), atazanavir (ATV), has a favourable adverse effect profile in comparison to lopinavir; therefore it has been included in the South African clinical guidelines for the management of HIV/AIDS in adults and adolescents (2010), as an alternative to lopinavir in patients who experience intolerable gastrointestinal problems, hyperlipidaemia or hyperglycemia [6]. Like other PIs, ATV is a substrate of the efflux transporter, P-gp [7, 8], which has a role to play in mediating absorption in the small intestine, as well as CYP3A4 and CYP3A5 [9] which facilitate metabolism in the small intestine and liver. ATV may thus be susceptible to pharmacokinetic (PK) interactions with agents able to modulate the activities of this transporter and family of CYP enzymes.

SF is a South African plant which has a long history of use in the practice of traditional medicine, particularly by the *isiZulu*, *isiSotho*, *isiXhosa,* and *Khoi-San* people [10, 11]. Anecdotal reports claim that SF may be useful for alleviating the cachexia (muscle-wasting) associated with HIV/AIDS [11]. Pharmaceutical dosage forms, such as tablets and capsules which contain milled SF leaves are now also available for purchase in pharmacies, health shops, and even online. Triterpenoid and flavonol glycosides (kaempferol and quercetin glycosides) have been isolated from SF plant material [12, 13] and are known to be present in several different samples of the plant [14]. In addition, SF is also purported to contain the non-protein amino-acid, L-canavanine, the inhibitory neurotransmitter, L-GABA, and the sugar, D-pinitol [11]. We recently conducted *in vitro* studies [15] which showed that aqueous extracts (10 mg/mL) of SF may have the potential to reduce ATV absorption and to inhibit ATV metabolism. A methanolic extract of SF in which less polar constituents in comparison to the aqueous extract are likely present may also inhibit ATV metabolism. A triterpenoid glycoside fraction isolated from SF enhanced absorption of the PI and enhanced its metabolism. These *in vitro* studies therefore alluded to the potential for traditional aqueous decoctions, as well as commercial preparations of SF to modulate the functional activity of ATV influx and/or efflux transporters, such as P-gp and ATV metabolic enzymes, such as CYP3A4 in the human small intestine and liver, respectively. However, the varying effects of the extracts and constituents of SF on ATV absorption and metabolism *in vitro* indicate that the true potential for SF to alter the bioavailability and PK of ATV may only be revealed by undertaking an *in vivo* study. Whilst ATV alone or unboosted is not used *per se* in the management of HIV/AIDs and ARV dosage regimens usually contain other classes of ARVs, the main objective of this study was to investigate the possibility of a drug-drug interaction between an ATM such as SF and ATV and to explore the implications thereof. An ATV single dose/SF multiple dose, one sequence crossover drug-drug interaction study in healthy male subjects was therefore conducted.

## 2. Materials and Methods

### 2.1. Materials

ATV sulphate (100.9%) was donated by Aspen Pharmacare (Port Elizabeth, South Africa), and diazepam (DIAZ) was obtained from the Biopharmaceutics Research Institute (Rhodes University, Grahamstown, South Africa). HPLC grade acetonitrile was purchased from Romil Ltd. (Cambridge, United Kingdom). Water was purified by reverse osmosis and filtration through a Milli-Q purification system (Millipore, Milford, M A, USA). Sodium carbonate (99.5%) and ethyl acetate (99–101%) were provided by BDH Laboratory Reagents (Poole, England) and formic acid (99.9%) from Associated Chemical Enterprises Pty Ltd. (Johannesburg, South Africa), whilst n-Hexane (≥98%) was purchased from Merck (Darmstadt, Germany). Fresh human plasma with potassium edetate (K-EDTA) as an anticoagulant was obtained from the South African National Blood Services, Eastern Cape Headquarters (Port Elizabeth, South Africa) and was stored at 4 ± 2°C. Medication used in the clinical study was Reyataz 200 mg ATV sulphate capsules from Bristol-Myers Squibb, Bedfordview, Gauteng, South Africa, and Sutherlandia SU1 300 mg SF tablets from Phyto Nova, Cape Town, Western Cape, South Africa. Analysis of these tablets revealed that triterpenoid glycosides, namely, Sutherlandiosides A, B, C, and D were present at 0.05, 3.02, 0.93, and 0.46 mg/tablet, respectively, while 0.63, 0.67, 1.49, and 0.99 mg/tablet of flavonol glycosides, namely, Sutherlandins A, B, C, and D respectively, were quantified.

### 2.2. Study Population

Twelve non-smoking, HIV-negative, healthy male subjects between the ages 18 and 55 years and with a body mass index (BMI) between 19 and 30 kg/m^2^ were enrolled into the study after giving informed consent and passing a medical, physical, and laboratory screening within one month prior to commencement of the study. The screening included laboratory tests for liver function, hepatitis B and C, and HIV, as well as blood biochemistry, urinalysis, and drugs-of-abuse, such as amphetamines, barbiturates, benzodiazepines, cocaine, methamphetamine, morphine, phencyclidine, tetrahydrocannabinol, tricyclic antidepressants, and alcohol.

The subjects were required to adhere to study restrictions which included no prescription or over-the-counter medicines from one week before the start of the study, no alcohol from four days before the start of the study, and no caffeine and grapefruit juice from 48 hours before the start of the study. Subjects were also prohibited from participating in strenuous exercise from 24 hours before each of the two phases of the study.

### 2.3. Study Design

Ethical approval was granted by the Rhodes University Ethical Standards Committee to conduct a one-sequence crossover, two phase clinical study with a single dose/multiple dose regimen combination for ATV and SF, respectively. The study was conducted according to the South African Good Clinical Trials guideline [16] and the Declaration of Helsinki and its amendments [17].

The night before the start of the study (Day 0), subjects checked into the clinic and were screened for drugs of abuse and probed to determine compliance with the study restrictions. On Day 1 of the study (start of Phase I), subjects received a light meal before a single 400 mg (2 × 200 mg capsules) dose of ATV (Reyataz, Bristol-Myers Squibb, Bedfordview, Gauteng, South Africa) was administered to each with a 240 mL glass of water. A mouth and hand check was conducted to confirm that the dose had been ingested. Subjects were not permitted to lie down or sleep until 4 hours after dose, unless this was necessary due to an adverse event. Standard meals were provided until 24 hours post-dose. The time at which each subject commenced and ended each meal was recorded as well as the approximate amount consumed.

Ten-millilitre blood samples were collected into BD Vacutainer blood collection tubes (Becton Dickinson, Woodmead, Gauteng, South Africa) containing potassium EDTA as the anticoagulant, at the following time intervals: before dosing (0) and at 0.5, 1.0, 1.5, 2.0, 2.5, 3.0, 3.5, 4.0, 5.0, 6.0, 9.0, 12, 18, and 24 hours post-dose. The exact time at which each sample was withdrawn was also recorded.

From Day 3, each subject started a twice daily regimen (one tablet twice a day) of Sutherlandia SU1 tablets (Phyto Nova, Cape Town, Western Cape, South Africa). The label of Sutherlandia SU1 stated that each tablet contained 300 mg of SF plant material. Subjects reported to the study investigator every day between 08h00 and 09h00 and between 20h00 and 21h00 to receive these doses with a 240 mL glass of water. A hand and mouth check was carried out to confirm that these doses were taken. On Day 14, the subjects checked into the clinic for screening as described for Day 0. On Day 15 (start of Phase II), each subject received a single oral dose of ATV (2 × 200 mg capsules) and a dose (1 × 300 mg tablet) of Sutherlandia SU1, 30 minutes after a light meal. The rest of Phase II was conducted according to the same procedures described for Phase I. Dropouts at any time during the study were not replaced.

Blood samples from both phases were stored in an ice-bath immediately after withdrawal up until centrifugation at 2800 ×g for 10 minutes at 4°C, which was done within 30 minutes. Duplicate aliquots of harvested plasma were stored in polypropylene tubes at −80 ± 10°C until transfer to the analytical laboratory, where the samples were stored at −10 ± 2°C. The tubes were labelled with the study number, phase number, subject number, sample number, and sampling time.

### 2.4. Safety and Tolerability

A pilot clinical study [18] has been conducted which was the first to provide a scientific basis for the safety of SF in healthy human subjects. Baseline medical and laboratory data were recorded before the study and were compared to post-study results of the tests conducted within three days of the last day of the study. During the study, blood pressure, pulse, and body temperature were monitored before and four hours after dosing with ATV. Subjects were also probed about their well-being using open-ended questions, and any adverse effects experienced were documented.

### 2.5. Analysis of Plasma Samples

The HPLC system consisted of an Alliance 2695 Separations module and a 2996 Waters photodiode array UV detector coupled to empower data acquisition software (Waters, Milford, MA, USA). A Luna C_18_ (2) (5 *μ*m, 150 × 2.0 mm ID) column (Phenomenex, USA) protected by a Luna C_18_ guard column (Phenomenex, USA) with the same ID was used to achieve chromatographic separation. The mobile phase was filtered under reduced pressure through a 0.45 *µ*m (PVDF) membrane (Durapore, Millipore, Bedford, MA, USA) and degassed using an Eyela Aspirator A-25 (Tokyo Rikakikai Co. Ltd., Tokyo, Japan) prior to use. A validated HPLC-UV method developed and validated in our laboratory [19] was used to analyse the plasma samples.

### 2.6. Non-Compartmental Analysis

The PK parameters of ATV before and after co-administration with SF were determined by non-compartmental analyses. Exposure measures, AUC_0–24 hr_ and *C*_max⁡_ were the primary PK parameters used to evaluate whether multiple dosing of SF altered the single-dose PK of ATV. Other parameters which were monitored included *t*_1/2_, *t*_max⁡_, and *k*_el_. These analyses were all conducted using the SAS software (SAS Institute Inc., Cary, NC, USA).

### 2.7. Statistical Analysis

Equiv Test (Statcon, Witzenhausen, Germany) was used to construct 90% CIs about the equivalence parameter and geometric mean ratio (difference of means on natural log scale) for AUC_0–24 hr_ and *C*_max⁡_. The ratio was computed as Phase II/Phase I, namely, ATV + SF/ATV alone. A clinically significant interaction was concluded if the 90% CIs for AUC_0–24 hr_ and *C*_max⁡_ were found to be outside the limits of 0.8–1.25. Based on the CIs for AUC_0–24 hr_ and *C*_max⁡_ and the number of subjects, an intra-subject CV% was estimated, which, with the geometric mean ratio for each systemic exposure, was utilised to compute the power of the study.

## 3. Results and Discussion

Of the 12 healthy male subjects enrolled to participate in the study, 8 (66.7%) were black, 3 (25%) were white, and 1 (8.3%) was Indian; the mean age was 23 years (range, 19–30 years) and an average BMI of 23.5 Kg/m^2^ (range, 20.2–27.6 Kg/m^2^) was recorded. The study was completed without any major protocol deviations. There were no subject dropouts and no adverse events were reported.

**Figure 1:**
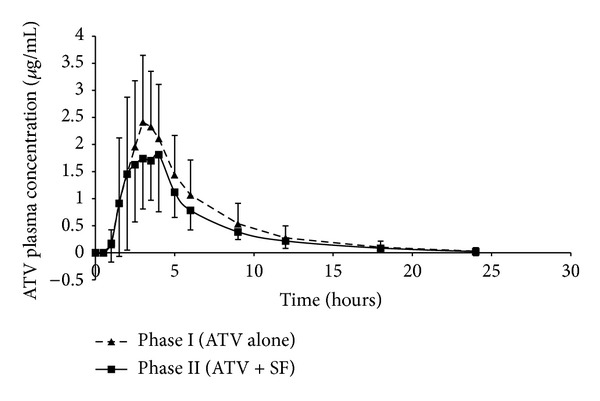
Comparison of ATV plasma concentration-time profiles for Phase I (ATV alone) and Phase II (ATV + SF). Each point represents the mean ± SD; *n* = 12.

Figure 1 shows the mean plasma concentration versus time profiles of ATV alone (Phase I) and ATV co-administered with SF (Phase II). It is evident that the two profiles were superimposable for the first 2 hours after dose. From 2 to 4 hours post-dose, the rate and extent of ATV absorption appeared to be reduced when ATV was co-administered with SF in comparison to ATV administered alone. From 4 to 24 hours post-dose, a similar rate of elimination was observed between the two phases. The statistical analysis (Table 1) revealed that for both *C*_max⁡_ and AUC_0–24 hours_, the geometric mean ratio (point estimate) and the lower limit of the 90% CI fell well below the lower limit of the “no-effect” boundary of 0.8–1.25, which suggests that a two-week regimen of Sutherlandia SU1 tablets significantly reduced the bioavailability of a single dose of ATV in this cohort of 12 healthy male subjects. Moreover, the power of the study was determined to be 95% and 93% when calculated from the *C*_max⁡_ and AUC_0–24 hr_ data, respectively. The sensitivity of the statistical approach applied to the data from 12 subjects was thus adequate to confirm that there was in fact a drug-drug interaction.

The significant reduction in the bioavailability of ATV, in the presence of SF may have occurred due to a decrease in absorption and/or enhanced metabolism of ATV. Potential mechanisms underlying the effect include an increase in the activities and/or the expression of influx and/or efflux transporters, such as P-gp and/or metabolic enzymes such as CYP3A4/5 in the small intestines and/or livers of some of the subjects. Protein expression via induction may only occur after chronic rather than acute administration of a xenobiotic, whilst modulation of the activity of the transporters and enzymes may manifest even after acute administration of the potential interacting agent. *In vitro* studies [15] have shown the potential for an aqueous extract of SF to reduce ATV absorption and for a triterpenoid glycoside fraction from SF to enhance ATV metabolism after acute exposure to the agents. In rats, a single dose of SF (12 mg/kg) had no significant effect on the PK parameters of a single dose of nevirapine [20], which may indicate that, *in vivo*, altered enzyme or transporter activity by SF may not have a major role to play in reducing the bioavailability of this NNRTI. However, ATV is a substrate of both P-gp and CYP3A4, whereas, nevirapine is only a substrate of CYP3A4. This indicates that modulation of the bioavailability of ATV by SF through altered transporter or enzyme activity may occur through both P-gp and CYP3A4 and may thus not necessarily exhibit the same effect as observed for nevirapine. On the other hand, chronic dosing of SF reduced the plasma levels of a single dose of nevirapine in rats, which correlated with an increase in the expression of CYP3A4 [20]. This effect may also have manifested to reduce plasma ATV levels after chronic dosing of SF in healthy humans. *In vitro* and *in vivo* studies to conclusively establish whether the PK interaction between ATV and SF is P-gp- and/or CYP 3A4-mediated should be conducted. The value of this lies in the potential to predict the role of genetic polymorphisms in the transporter and/or enzyme involved in the interaction.

The similar rate of elimination observed between the two phases from 4 to 24 hours post-dose may indicate that SF did not alter post-absorption metabolism or transport pathways of ATV which occur as part of the elimination process in the liver and that the observed decrease in bioavailability of ATV was more likely due to a reduction in the transport and/or an increase in metabolism of ATV during the absorption process in the small intestine. This may imply that the change in activity and/or expression of transporters and/or metabolic enzymes in the small intestines was greater than in the livers of susceptible subjects. To exert an effect in the liver, the “active” phytochemical constituents of SF must be absorbed across the intestinal epithelium into the systemic circulation. The absorption of at least one of the triterpenoid glycosides present in SF may be impeded, since *in vitro* studies demonstrated that it is subject to P-gp-mediated efflux in Caco-2 cells [21]. Similarly, the absorption of other SF constituents may also be hindered; therefore the concentrations of these which reach the liver may not be sufficiently high to exert the effect. This hypothesis needs to be investigated further by application of PK modelling to the data.

The clinical relevance of the potential interaction between SF and ATV is difficult to predict since only a single dose of ATV was evaluated, yet clinically, ATV is dosed chronically. It is therefore not known how SF may affect the steady-state PK of ATV, and thus whether subtherapeutic levels of ATV may result. The bioavailability of ATV is reduced in HIV patients in comparison to healthy subjects [22]; therefore the effect may be more pronounced and severe in patients. On the other hand, the effects of SF may also be diminished if ritonavir, a CYP3A4 inhibitor, is co-administered with ATV as a booster in ARV-experienced patients [22] Moreover, other covariates such as comorbidities and comedication, which also influence enzyme and/or transporter activity or expression [23] were not considered.

The PK of ATV has been found to be influenced by CYP3A5 and P-gp genetic polymorphisms [24]. CYP3A5 expressors had a faster clearance which resulted in a lower *C*_min⁡_ of ATV than non-expressors. Individuals who possess the wild-type ABCB1 (P-gp) CGC haplotype had a slower clearance of ATV. The primary implication of CYP3A5 polymorphism for ATV-drug PK interactions is that CYP3A5 non-expressors may be more susceptible to CYP3A4-mediated interactions, whether through altered activity or expression, since these individuals rely entirely on CYP3A4 for metabolism of ATV. The association of wild-type ABCB1 (P-gp) CGC haplotype with a slower clearance may suggest that, in individuals who possess this P-gp genetic polymorph, P-gp-mediated transport of ATV is less significant compared to those who exhibit other P-gp polymorphs. These individuals may therefore be at a lower risk of ATV-drug interactions mediated through P-gp. If studies to determine the mechanism by which SF alters the bioavailability of ATV reveal that the effect is mediated by P-gp and/or CYP3A4, then the potential for this ARV-ATM interaction in a population may be largely governed by the predominance of certain genotypes of the transporter and/or enzyme. In the present study, the effect of SF on the bioavailability of ATV was more significant in a few individuals in comparison to the majority. This suggests that genetic polymorphisms of the transporters and/or enzymes involved may have had a role to play in aggravating or mitigating the effect.

A significant decrease in the steady state *C*_max⁡_ and AUC of other PIs, indinavir and saquinavir, has been demonstrated when these were coadministered with complementary medicines (CAMs), St. John's wort (indinavir) [25], vitamin C (indinavir) [26], and garlic (saquinavir) [27] to ≤10 healthy subjects, also in one-sequence crossover studies. The approximately 20% decrease in ATV *C*_max⁡_ and AUC_0–24 hr_ by SF in this study was comparable to the effect of vitamin C on indinavir but not as significant as the effects of St. John's Wort and garlic on indinavir and saquinavir, respectively, where up to a 57% decrease in one of the PK parameters was observed. These studies in the literature, together with the findings of this study highlight the potential susceptibility of the PIs to PK interactions which result in reduced bioavailability. The need for health care providers to be aware of the CAMs and/or ATMs which patients may be using is therefore emphasised so that (i) preclinical and clinical data on the potential for PK interactions between specific PIs and CAMs or ATMs known to be used concomitantly may be generated and (ii) so that patients may be advised accordingly to prevent clinically proven interactions between CAMs or ATMs and the PIs from occurring.

## 4. Conclusions

A two-week regimen of Phyto Nova Sutherlandia SU1 tablets which contain SF plant material significantly reduced the *C*_max⁡_ and AUC_0–24 hr_ of a single dose of ATV in healthy male subjects, implying that the bioavailability of ATV may be reduced in the presence of SF. SF and ATV co-administration may thus potentially result in subtherapeutic plasma levels of ATV which may in turn cause ATV resistance and treatment failure. Clearly, the presence of other classes of ARVs used in dosing regimens and possible effects during concurrent therapy with ATMs requires investigation. This research has highlighted the potential risk for a reduction in efficacy of an ARV regimen which may ocurr when ATMs and PIs are used concurrently and that patients and health care practitioners alike should be aware of these perils.

## Figures and Tables

**Table 1:** Non-compartmental and statistical analysis of PK parameters of ATV.

Pharmacokinetic parameter	Phase I (ATV alone) Arithmetic mean (CV%)	Phase II (ATV + SF) Arithmetic mean (CV%)	Phase II/Phase I Geometric mean ratio (90% CI)
AUC_0–24_ (*µ*g/mL·hour)	13.0 (50.3)	10.0 (38.6)	0.801 (0.634–1.01)
*C*_max⁡_ (*µ*g/mL)	3.17 (30.3)	2.59 (43.6)	0.783 (0.609–1.00)
*T*_max⁡_ (hours)	2.71 (26.7)	2.67 (33.3)	N/A
*T*_1/2_ (hours)	3.77 (39.5)	3.82 (35.1)	N/A
*k*_el_ (hour^−1^)	0.205 (31.2)	0.200 (30.9)	N/A
